# Extranodal Ocular Adnexal Marginal Zone Lymphoma in a Ten-Year-Old Child

**DOI:** 10.4274/tjo.galenos.2019.62592

**Published:** 2020-03-05

**Authors:** Nazan Çetingül, Melis Palamar, Şükriye Hacıkara, Serra Kamer, Hamiyet Hekimci Özdemir, Eda Ataseven, Özlem Barut Selver, Mine Hekimgil

**Affiliations:** 1 Ege University Faculty of Medicine, Department of Child Diseases, İzmir, Turkey; 2 Ege University Faculty of Medicine, Department of Ophthalmology, İzmir, Turkey; 3 Ege University Faculty of Medicine, Department of Radiation Oncology, İzmir, Turkey; 4 Ege University Faculty of Medicine, Department of Patalogy, İzmir, Turkey

**Keywords:** Conjunctiva, eye, lymphoma, marginal zone, ocular adnexal lymphoma

## Abstract

A 10-year-old girl was brought to the clinic with the complaint of a salmon-colored conjunctival lesion for 1 month. With the aid of histopathological evaluation and other tests, extranodal ocular adnexal marginal zone lymphoma was diagnosed. The patient was graded as T1bN0M0 according to AJCC and Stage 1 according to Ann Arbor classification. She was treated with external radiotherapy at 1.8 Gy/day for 17 days for a total dose of 36 Gy. She is in remission for 26 months and still being followed up.

## Introduction

Lymphomas in the ocular adnexa predominantly result from B-cell proliferation and can arise from the conjunctiva, eyelids, lacrimal glands, and orbit. The most common type of ocular adnexal lymphoma (OAL) is extranodal marginal zone B-cell lymphoma (MZL), which is an extremely rare subtype of non-Hodgkin lymphoma (NHL) in childhood.^1,2^ Although most NHL in children show aggressive behavior, this rare type tends to be indolent. There are few reported cases of primary ocular adnexal MZL in children.^3^ OALs have recently been classified according to the American Joint Committee on Cancer (AJCC) stating system with variable prognoses.^4^

Some infectious agents, immunodeficiency, autoimmunity, genetic mutations and immunosuppression have been linked to MZL and appear to be integral to the etiopathology.^5^ Retrospective studies documented the use of various treatment modalities such as local surgery alone, local radiotherapy alone, and several chemotherapy, targeted therapy, immunotherapy, or antimicrobial therapies in adult patients. Although local radiation may provide local control in adults, there is no well-established treatment modality for pediatric patients.^6^

## Case Report

**Figure 1:**
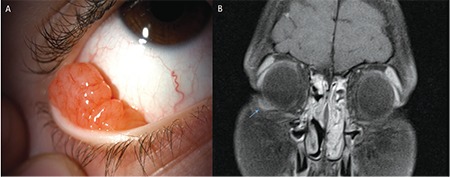
A) The salmon-colored lesion at the inferotemporal conjunctiva. B) The mass lesion on the right inferolateral region on T1 fat-suppressed MRI (arrow)

**Figure 2:**
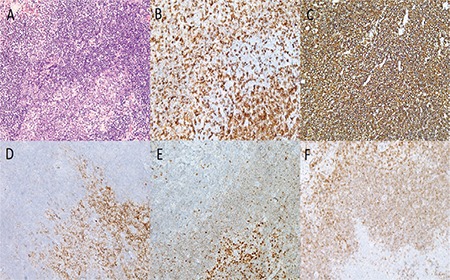
A) Neoplastic small lymphoid cell infiltration and follicular colonization (residual germinal center on the right lower field) (H&E, x20). B) CD3 was expressed on the rare nonneoplastic T lymphocyte population. C) CD20 positivity of neoplastic B lymphocytes. D, E.) CD10 and Bcl-6 positivity of residual germinal center cells. F) Bcl-2 negativity of residual germinal center cells and positivity of neoplastic B lymphocytes (B-F- DAB, x20)

A 10-year-old girl presented to the ophthalmology department with a fast-growing salmon-colored mass protruding from the medial aspect of the right lower eyelid for approximately 1 month (Figure 1A). The orbital lesion was also demonstrated on magnetic resonance imaging (MRI) (Figure 1B) and excisional biopsy of the mass was performed. Histopathological examination revealed B-cell MZL (Figure 2A, B, C, D, E, F).

Upon diagnosis, the patient was referred to the pediatric oncology department. On physical examination, lymphadenopathy and hepatosplenomegaly were not detected. The metastatic workup including hemogram, biochemistry tests, immunoglobulins, brain MRI, cerebrospinal fluid cytology, and bone marrow study were within normal limits. Autoantibody tests were negative. *Helicobacter pylori*, *Chlamydia psittaci*, and *Chlamydia trachomatis* antibodies were negative. Conjunctival smear was unremarkable. The patient did not have any history of conjunctivitis or exposure to birds. Apart from the lesion in the right eye, no other pathological involvement was detected in PET-CT. The patient was graded as T1bN0M0 according to AJCC and as Stage 1 according to Ann Arbor staging.

As repeated ophthalmological examinations revealed bilateral suspicious follicular reaction, *Chlamydia* was assumed and antimicrobial treatment (doxycycline, 200 mg/day) was initiated. After 2 weeks of treatment, progression of the tumor was observed, and external radiotherapy was planned immediately. The prescribed dose was 36 Gy in 17 fractions to the isocenter (1.8 Gy/fraction dose) using 6 MeV electrons. Cerrobend block was created to protect the lens. Radiotherapy resulted in rapid remission. The child is currently in remission for 26 months. The patient’s mother consented to all treatments and publication of this article.

## Discussion

Pediatric B-cell MZLs are detected in the marginal zone of secondary lymphoid tissues. These lymphomas can occur in lymph organs/nodes (nodal MZL) (15%), spleen (15%), and in nonlymphoid organs such as conjunctiva, lung, skin, stomach, orbit, and dura (extranodal MZL) (70%).^1,2,5^ Pediatric extranodal MZL is an extremely rare entity, comprising less than 1% of all pediatric lymphomas.^1,2,5^

Extranodal MZLs that occur in the periocular region are termed as OAL and often involve several tissues such as conjunctiva, orbit, and eyelid. The incidence of OAL is approximately 0.2 per 100,000 individuals. Most OALs are B-cell NHL belonging to 1 of 5 subtypes: extranodal marginal zone/mucosa-associated lymphoid tissue (MALT) lymphoma, follicular lymphoma, diffuse large B-cell lymphoma, mantle cell lymphoma, and lymphoplasmacytic lymphoma.^7^ Extranodal MZL/MALT lymphomas are approximately 80% low grade and typically follow an indolent course.

MZL is a relatively common entity in adults (5-17% of all NHL diagnoses). The median age at MALT/MZL diagnosis is approximately 60 years, and few pediatric cases have been reported.^2,7^ Some chronic infectious agents (H. pylori in gastric extranodal MZL, *Chlamydia* spp. in ocular adnexal MZL, etc.), immunodeficiency, autoimmunity (Sjögren’s syndrome, Hashimoto’s thyroiditis, and other autoimmune diseases), genetic mutations, and immunosuppression have been reported to be linked to MZL.^7^ Chronic inflammation at the affected site certainly plays a major role, with both exogenous and endogenous antigens being potentially responsible for the activation of inflammatory responses in ocular adnexal MZL patients.^5^ In the present 10-year old patient, *H. pylori*, *Ch. psittaci*, and *Ch. trachomatis *antibodies and all autoimmune tests were negative and conjunctival smear was unremarkable.

Morphologically, extranodal MZL resembles the adult counterparts, and the differential diagnosis includes reactive marginal zone hyperplasia and pediatric follicular lymphoma. These blastic cells from the diagnostic lymphoepithelial lesions are helpful in the morphologic distinction of this lymphoma from reactive lymphoid hyperplasia associated with chronic inflammation such as chronic conjunctivitis. The blastic cells express the common B cell markers such as CD20.^8^ The present case presented dense small lymphocytic infiltrate with destruction of normal architecture, forming follicular colonization. Immunophenotypically, the neoplastic cells were positive for CD20, BCL2, CD23, and CD43, but negative for CD5, CD10, CD23, BCL6, and cyclin D1. The diagnosis was confirmed by the BCL2 and CD43 expression of neoplastic small lymphocytes, which may further be confirmed by the demonstration of monotypical expression of light chains on the plasma cell component by immunohistochemistry or in-situ hybridization. Unfortunately, the plasma cells were scarce in our case, and showed polytypical expression of both the light and heavy chains by immunohistochemistry. Monoclonal IgH rearrangement might be demonstrated by PCR to further confirm the clonal nature of the infiltration, which was not performed in our case due to the small amount of tissue left. Bilateral bone marrow biopsies and aspirates did not reveal any neoplastic infiltration. As no other pathological involvement was detected on PET-CT, the patient was diagnosed as T1bN0M0 according to AJCC and Stage 1 according to Ann Arbor classification.

Primary localized ocular adnexal MZLs are malignancies that show indolent behavior, usually associated with a favorable prognosis. Only a few large prospective studies on the effectiveness of the various treatment options can be found in the literature. For ocular adnexal MZLs of the conjunctiva in adults, treatment is generally planned according to stage of the disease. Surgery or radiotherapy has been used in the management of adult patients with low-grade ocular adnexal MZLs of the conjunctiva. The few reported cases of conjunctival extranodal MZLs in the pediatric population were all stage 1 disease. For the treatment of stage 1 ocular adnexal MZLs, a decision must be made between surgical excision or antimicrobial therapies and/or radiotherapy, or immunotherapy.^9,10^ Currently the role of surgery is limited only to diagnostic biopsy. In a recent study reporting the outcomes of 71 newly diagnosed ocular adnexal MZL patients treated with radiotherapy (median dose of 30 Gy in 15 fractions), local control of the disease was obtained in 100% of the patients.^11^ Toxicity was acceptable and limited to grade 1 acute conjunctivitis (20%) and acute erythema (20%), with only 4% of patients developing acute dry eye. However, late cataract was observed in 6%. As repeated examinations of the present patient showed follicular reaction in bilateral conjunctival regions, chlamydia was assumed and antimicrobial treatment (doxycycline 200 mg/day) was initiated. In the third week of treatment, the tumor enlarged rapidly. For this reason, external radiotherapy 1.8 Gy/day for 7 days with a total dose of 36 Gy was performed. No complications other than acute erythema were observed due to radiotherapy and the patient is currently in remission for 26 months.

In conclusion, although rare, ocular adnexal MZL may also occur in children. As with adult patients, biopsy is mandatory to achieve prompt diagnosis in suspected cases. Systemic evaluation for additional involvement should also not be neglected.
